# Integration of Metabolomics and 16S Ribosomal RNA Sequencing to Elucidate the Pathogenesis of Ankylosing Spondylitis

**DOI:** 10.1002/iid3.70183

**Published:** 2025-03-26

**Authors:** Xin Wang, Haojie Xu, Yuyan Chao, Chao Sun, Tingting Wang, Xiaoyun Fan, Lin Tang, Shengqian Xu, Changhao Xie

**Affiliations:** ^1^ Department of Rheumatology and Immunology The First Affiliated Hospital of Bengbu Medical University Bengbu China; ^2^ Department of Rheumatology and Immunology The First Affiliated Hospital of Anhui Medical University Hefei China; ^3^ Anhui Province Key Laboratory of Immunology in Chronic Diseases Bengbu China; ^4^ Anhui Province Key Laboratory of Basic and Translational Research of Inflammation‐Related Diseases Bengbu China; ^5^ Department of Rheumatology and Immunology Peking University People's Hospital Beijing China; ^6^ Department of Rheumatology and Clinical Immunology Peking Union Medical College Hospital, Chinese Academy of Medical Sciences/Peking Union Medical College Beijing China; ^7^ Department of Medical Research Center National Science and Technology Key Infrastructure on Translational Medicine, Peking Union Medical College Hospital, Chinese Academy of Medical Sciences/Peking Union Medical College Beijing China; ^8^ Biomarker Technologies Corporation Beijing China

**Keywords:** 16S rRNA sequencing, ankylosing spondylitis, blood metabolome, gut microbiota, metabolomics

## Abstract

**Objective:**

Despite growing interest in the gut microbiota and blood metabolome in patients with ankylosing spondylitis (AS), its role remains poorly understood. Here, we investigate how microbial and metabolic alterations contribute to AS.

**Methods:**

Fecal microbiome data from 40 AS patients were compared with those from 40 healthy controls (HCs) using 16S ribosomal RNA (rRNA) gene sequencing. The plasma metabolic profiles were analyzed and integrated with the microbiota data to identify biological characteristics specific to AS.

**Results:**

AS patients showed significant enrichment of specific genera, including *Megamonas, Elusimicrobium, Dysgonomonas, Ruminococcus_gauvreauii_group*, and *unclassified_Prevotellaceae*. Pathways with the most differentially expressed metabolites included bile secretion; neomycin, kanamycin, and gentamicin biosynthesis; and arachidonic acid metabolism. Positive correlations between *Megamonas* and *Elusimicrobium* and metabolites such as piribedil, l‐cystathionine, and crocetin dialdehyde suggested microbial enrichment in AS patients.

**Conclusions:**

A disrupted gut microbiota and altered metabolites are present in AS patients. Integrating microbiome and metabolomic data reveals significant disruptions in AS patients, improving our understanding of its pathogenesis.

## Summary

- *What is already known on this topic*—Little is known about the relationship between changes in the gut microbiota and metabolome in patients with ankylosing spondylitis (AS).
- *What this study adds*—AS patients presented a disrupted gut microbiota and altered metabolites. The analysis of microbiome and metabolomic data revealed significant differences in the gut microbiota and metabolites between AS patients and healthy controls (HCs).
- *How this study might affect research, practice, or policy*—These preliminary findings provide valuable insights into the potential pathogenesis of AS, which warrants further exploration through multiomic studies with larger data sets.

## Graphical abstract

Disrupted gut microbiota and altered metabolites are present in AS patients. The analysis of microbiome and metabolomic data revealed significant differences between AS patients and HCs.

## 1 Introduction

Ankylosing spondylitis (AS) is a chronic inflammatory disease that primarily affects the sacroiliac joint, spine, paraspinal soft tissue, and peripheral joints and can lead to spinal deformity and rigidity [1, 2]. Subclinical intestinal inflammation occurs in 40%–60% of AS patients, with progression to inflammatory bowel disease (IBD) in 5%–10% of patients [2]. Furthermore, up to 44% of IBD patients experience inflammatory arthritis, suggesting a possible common pathogenesis between the two conditions [3, 4]. The exact etiology of AS remains unclear. Among the possible factors, the roles of the gut microbiota and metabolome have become a focal point of interest [5].

Metabolites are critical for understanding disease processes, as they reflect the metabolic state of the body and can directly influence immune responses, inflammation, and cellular functions. In autoimmune diseases such as rheumatoid arthritis (RA), systemic lupus erythematosus (SLE), and Behçet's disease (BD), altered metabolite profiles have been linked to the disease and disease activity. Metabolites such as short‐chain fatty acids (SCFAs), amino acids, and lipids can interact with immune cells, modulating immune function and inflammation. Changes in metabolites and microbial products can drive chronic inflammation, suggesting that they may play a key role in the onset and progression of diseases like AS.

AS patients exhibit abnormally elevated levels of interleukin‐23 (IL‐23) in their intestines [6]. IL‐23 plays a crucial regulatory role in mucosal immunity, mediating antimicrobial responses and extracellular defences on epithelial surfaces [7]. IL‐23 promotes T helper 17 (Th17) cells proliferation and interleukin‐17 (IL‐17) secretion, driving downstream inflammatory responses in AS patients [8, 9].

A healthy gut microbiota plays a vital role in defending against pathogenic microbes invading mucosal epithelial cells and the blood circulation, forming a critical physiological basis for the function of the mucosal barrier [10, 11]. Alterations in the gut microbiota can trigger persistent antigen stimulation, activating T cells and causing chronic inflammation [12, 13]. An imbalance in the gut microbiota leads to pathogen proliferation and changes in the microbial distribution, which reduce mucosal surface permeability and impair barrier function [14]. Pathogens that penetrate the mucosa activate innate immunity and produce various proinflammatory factors [15]. Disruptions in the gut microbiota and plasma metabolome, characterized by significant differences compared with those in healthy individuals, have been observed in patients with several autoimmune diseases, such as RA, SLE, and BD [6, 16, 17]. Alterations in metabolites and the gut microbiota composition have also been reported in AS patients, suggesting a shared pathophysiological link between microbial dysbiosis, metabolic disturbances, and autoimmune diseases. Similar evidence from individuals with other spondyloarthritis (SpA) subtypes, such as reactive arthritis, has also indicated the involvement of an imbalanced gut microbiota [18]. A study of reactive arthritis revealed that joint synovial CD8+ T cells could recognize the recombinant outer membrane protein A from *Salmonella*, stimulating mononuclear cells in the synovial fluid to produce IL‐23 and IL‐17, suggesting that pathogenic microbes can induce immune inflammatory responses that lead to spondylitis [19].

Recent studies have reported an overgrowth of specific bacterial genera in the gut of AS patients, including *Bacillus anthropophilus*, *Bifidobacterium bifidum*, *Acidaminococcus fermentans*, and *Prevotella* sp., indicating a potential link between an imbalanced gut microbiota and AS development [20, 21]. Associations between altered blood metabolites and AS have also been reported [20]. Various cellular and molecular mechanisms, including aberrant microbial translocation, antigen mimicry, and disrupted immune responses influenced by microbial metabolites, have been proposed to explain how the gut microbiota and metabolites contribute to autoimmunity [5, 22]. Studies have shown that specific metabolites produced by gut bacteria can modulate immune responses, further contributing to the inflammatory environment observed in AS patients. AS patients and their first‐degree relatives exhibit increased gut permeability, which increases their exposure to gut microbes [23]. However, the correlations between these factors and AS remain largely unclear, with a lack of definitive explanations [24, 25].

Another critical aspect of the role of IL‐23 is the interaction between the gut microbiota and AS. Studies in germ‐free environments with HLA‐B27 transgenic mice that do not exhibit characteristics of SpA suggest that merely being HLA‐B27‐positive may not be sufficient to progress to AS [26, 27]. Another study conducted under germ‐free conditions revealed slower disease progression in SpA mice, along with reduced ileal IL‐23 expression [28]. These studies suggest that microbial alterations might be a natural process in the disease progression of AS, independent of genetic traits [29]. Investigating the composition of the gut microbiota and its metabolomic characteristics in AS patients may provide further insights into the mechanisms of this disease [21].

In this study, we performed a comprehensive analysis of the microbiological and metabolomic profiles of AS patients to identify specific microbial and metabolic signatures associated with this disease. Our findings highlight significant alterations in both the gut microbiota composition and the plasma metabolome in AS patients, including the overgrowth of specific bacterial genera and the presence of distinct metabolites. These findings suggest a potential association between dysbiosis and AS progression, though causality requires further validation. Moreover, we observed that certain metabolic pathways influenced by gut microbial metabolites were significantly associated with inflammatory markers and disease severity in AS patients. Through a detailed analysis, this study explored how the gut microbiota and metabolites influence immune responses in AS patients. These findings could pave the way for novel therapeutic strategies targeting the gut microbiota and metabolic pathways, offering more personalized approaches for managing AS.

## 2 Materials and Methods

### 2.1 Study Design

Forty AS patients who responded poorly to two different nonsteroidal anti‐inflammatory drugs (NSAIDs) were recruited from The First Affiliated Hospital of Bengbu Medical University (BBMU). These patients had not received glucocorticoids, antibiotics, conventional synthetic disease‐modifying antirheumatic drugs (csDMARDs), biologic DMARDs (bDMARDs), or targeted synthetic DMARDs (tsDMARDs) in the past 3 months. All patients fulfilled the modified New York criteria for AS from 1984 [30]. Disease activity was evaluated using the AS Disease Activity Index based on C‐reactive protein (ASDAS‐CRP), Bath AS Functional Index (BASFI), Bath AS Metrology Index (BASMI), Patient Global Assessment (PGA), AS Quality of Life (ASQoL), and Patient Back Pain Intensity Assessment (PBPIA). Age‐ and sex‐matched individuals were included as healthy controls (HCs). The exclusion criteria included individuals who had received antibiotics, glucocorticoids, csDMARDs, bDMARDs, or tsDMARDs within the past 3 months, as well as those with severe systemic diseases, acute or chronic inflammatory or infectious diseases, or autoimmune conditions. Clinical data were collected within 3 days prior to the collection of fecal and blood samples.

### 2.2 Sample Collection and Processing

After defecation, patients used a scoop to collect a sample from the inner core of the stool. This technique minimizes the exposure of microbial communities to oxygen, although it cannot entirely eliminate exposure [31]. The collected fecal sample was then transferred into a sterile storage tube containing a fecal bacteria preservation solution, RNAlater (Thermo Fisher Scientific, Waltham, MA, USA). It is a nontoxic aqueous solution that inhibits bacterial growth and preserves the sample without the need for liquid nitrogen. It rapidly permeates tissues, stabilizes and protects cellular RNA and prevents DNA degradation, making it an effective preservative for fecal samples. The samples were transferred to a −80°C freezer within 2 h. Once all the samples were collected, they were transported to the laboratory on dry ice for analysis [32, 33]. We extracted DNA using the TGuide S96 Magnetic Soil/Stool DNA Kit (Tiangen Biotech). DNA purity and integrity were assessed using agarose gel electrophoresis. Samples with an optical density ratio (260/280 nm) between 1.8 and 2.0 and DNA levels > 1 μg were used for library construction.

For blood samples, 20 μL of human plasma was added to a 96‐well plate and transferred to an Eppendorf epMotion Workstation (Eppendorf Inc., Humburg, Germany). A total of 120 μL of internal standards was added, and the samples were vigorously vortexed for 5 min. After centrifugation at 4000*g* for 30 min, the supernatant was transferred to a clean 96‐well plate. Finally, 20 μL of freshly prepared derivative reagents was added, and the samples were incubated at 30°C for 60 min before analysis.

### 2.3 16S Ribosomal RNA (rRNA) Gene Amplicon Sequencing Analysis

After total DNA was extracted from the samples, the V3‒V4 region of the 16S rRNA gene was amplified using the universal primers 338F/806R (5′‐ACTCCTACGGGAGGCAGCAG‐3′, 806R 5′‐GGACTACGVGGGTWTCTAAT‐3′) [34]. PCR amplification was performed in a total reaction volume of 10 μL, with DNA template amounts ranging from 5 to 50 ng and primers at 10 μM concentrations. The amplification conditions included initial denaturation at 95°C for 5 min, followed by 20 cycles of denaturation at 95°C for 30 s, annealing at 50°C for 30 s, and extension at 72°C for 40 s, with a final extension step at 72°C for 7 min. The PCR products were purified using an Omega DNA purification kit and quantified with a Qsep‐400 system. Sequencing was subsequently performed on the Illumina NovaSeq. 6000 platform with paired‐end reads (2 × 250) after quality control was performed using Trimmomatic v0.33. Clean reads were processed using dada2 to generate amplicon sequence variants (ASVs) [35], and any ASVs with a relative abundance of < 0.005% across all samples were filtered out. The taxonomic classification of the ASVs was performed using the naive Bayes classifier in QIIME2, with reference to the SILVA database (Version 138.1) [36] and a confidence threshold of 70%, ensuring precise and standardized taxonomic assignments. Both alpha and beta diversity metrics were calculated using QIIME2 to assess microbial diversity [37]. Principal coordinate analysis was employed to visualize the beta diversity among samples. Furthermore, taxonomic differences among groups were analyzed using linear discriminant analysis (LDA) effect size (LEfSe), with a logarithmic LDA score threshold of 2.0, to identify microbial features that discriminated between groups.

### 2.4 Metabolomic Analysis

The untargeted metabolomic analysis was conducted using a liquid chromatography quadrupole time‐of‐flight (LC‐QTOF) mass spectrometer to explore the metabolite profile in AS patients. The quality control samples exhibited tight clustering in the principal component analysis, indicating excellent system stability. The data were normalized by adjusting the peak areas with respect to the total peak area. Spearman's correlation analysis was employed to assess the repeatability of samples within groups and the quality control samples.

The identified metabolites were further annotated by querying the KEGG, HMDB, and LipidMaps databases for classification and pathway analyses. Differentially abundant metabolites were determined based on fold change (FC) > 1, *p* value < 0.05, and VIP value > 1. Orthogonal partial least squares discriminant analysis (OPLS‐DA) was performed using the R package ropls with 200 permutations to verify the model's reliability and to ensure the robustness of the findings. Additionally, a hypergeometric distribution test was employed for the KEGG pathway enrichment analysis of the differentially abundant metabolites to identify significant metabolic pathways.

### 2.5 Statistical Analysis

Descriptive statistics were calculated for all the variables. Continuous data are presented as the means ± standard deviations (SDs) for normally distributed data or as medians with interquartile ranges for nonnormally distributed data. The distribution of the data was assessed visually using histograms and quantitatively via the Shapiro‒Wilk test. For pairwise comparisons, the Mann‒Whitney *U* test was used to analyze nonnormally distributed data, whereas Student's *t* test was employed for normally distributed data. These tests were selected based on the results of normality testing and were applied to assess differences in the microbiome and metabolomic data between HCs and AS patients.

LEfSe was performed to identify significant differences in the gut microbiota between the HC group and AS patients. The LDA score threshold was set at > 2 to determine the gut microbiota features with the most significant differential abundance between the two groups. LEfSe was performed using the default settings within LEfSe software, with all the statistical tests adjusted for multiple comparisons using the Benjamini‒Hochberg method to control for the false discovery rate (FDR).

For the metabolomic analysis, the differentially abundant metabolites were screened using OPLS‐DA. Metabolite variables contributing significantly to the model were identified based on a variable importance in projection (VIP) score threshold of > 1 and a Mann‒Whitney *U* test threshold of *p* < 0.05. For this analysis, cross‐validation was applied to ensure the robustness of the OPLS‐DA model. All the statistical analyses were performed using R and IBM SPSS Statistics, Version 26.0 (IBM Corp., Armonk, NY, USA).

Spearman's rank correlation analysis was conducted to evaluate the relationships between key clinical variables (e.g., ASDAS, BASFI, and BASMI) and the gut microbiota or metabolite abundances. The correlation coefficients were computed, and statistical significance was determined at *p* < 0.05.

The raw data generated by MassLynx V4.2 software underwent peak extraction and alignment using Progenesis QI software V2.3. The data were preprocessed to ensure the removal of noise and interference, and only peaks with a signal‐to‐noise ratio > 3 were included in the final analysis. Metabolites were identified by comparing their mass spectra with reference data from the METLIN database from Biomarker Technologies Corporation (Beijing, China). The identification of theoretical fragment ions was performed with a mass error tolerance within 100 ppm.

All analyses were performed with a two‐sided significance level of *p* < 0.05. For multiple comparisons, adjustments were made using the FDR when appropriate.

## 3 Results

### 3.1 Clinical Characteristics of AS Patients

This study included 40 participants who were diagnosed with AS and 40 in the HC group. Basic demographic characteristics, including sex (both groups: females 9/40, 22.50%), age (AS: median 33.00 years, [IQR 30.00, 43.25]; HC: median 32.00 years [IQR 27.00, 42.75]), and body mass index (BMI) (AS: mean 24.48 kg/m^2^, SD 22.54–26.30; HC: 23.56 kg/m^2^, SD 21.48–26.96), were not significantly different between the two groups. The demographic data for the HC group and the clinical characteristics of the AS patients are presented in Table 1.

**Table 1:** Clinical data for the enrolled ankylosing spondylitis patients.

	AS patients (*N* = 40)	HC group (*N* = 40)
Female (%)	9 (22.50%)	9 (22.50%)
Age at AS onset (years, median [IQR])	24.00 (19.25, 32.75)	—
Duration (years, median [IQR])	9.50 (4.25, 11.00)	—
Age at enrollment (years, median [IQR])	33.00 (30.00, 43.25)	32.00 (27.00, 42.75)
Body mass index (kg/m^2^, mean ± SD)	24.48 (22.54, 26.30)	23.56 (21.48, 26.96)
Assessment of the pelvis on plain film radiography	Bilateral level 2 (10, 25.00%) Bilateral level 3 (19, 47.50%) Bilateral level 4 (6, 15.00%) Right side level 3, left side level 1 (1, 2.50%) Right side level 4, left side level 3 (1, 2.50%) Right side level 2, left side level 3 (1, 2.50%) Right side level 3, left side level 2 (2, 5.00%)	
HLA‐B27 (positive, %)	36 (90.00%)	0
ESR (mm/h)	12.00 (6.00, 28.75)	
CRP (mg/L)	14.78 (5.79, 21.37)	
Patients' back pain intensity assessment	6.00 (5.00, 6.50)	
PGA	6.00 (5.00, 7.00)	
BASDAI	5.65 (5.01, 6.15)	
BASFI	4.80 (3.60, 5.65)	
ASQoL	10.00 (7.00, 11.75)	
PhGA	6.00 (5.00, 6.00)	
BASMI	4.50 (1.25, 6.00)	
ASDAS‐CRP	3.54 (3.03, 3.88)	
ASDAS‐CRP < 1.3 (low activity)	0	
1.3 ≤ ASDAS‐CRP < 2.1 (medium activity)	1 (2.50%)	
2.1 ≤ ASDAS‐CRP < 3.5 (high activity)	19 (47.50%)	
ASDAS‐CRP ≥ 3.5 (very high activity)	20 (50.00%)	

Abbreviations: ASDAS, AS Disease Activity Score; ASQoL, AS Quality of Life; BASDAI, Bath AS Disease Activity Index; BASFI, Bath AS Functional Index; BASMI, Bath AS Metrology Index; CRP, C‐reactive protein; ESR, erythrocyte sedimentation rate; IQR, interquartile range; PGA, Patient Global Assessment; PhGA, Physician Global Assessment; SD, standard deviation.

### 3.2 Analysis of the Diversity of the Gut Microbiota in the AS and HC Groups

**Figure 1:**
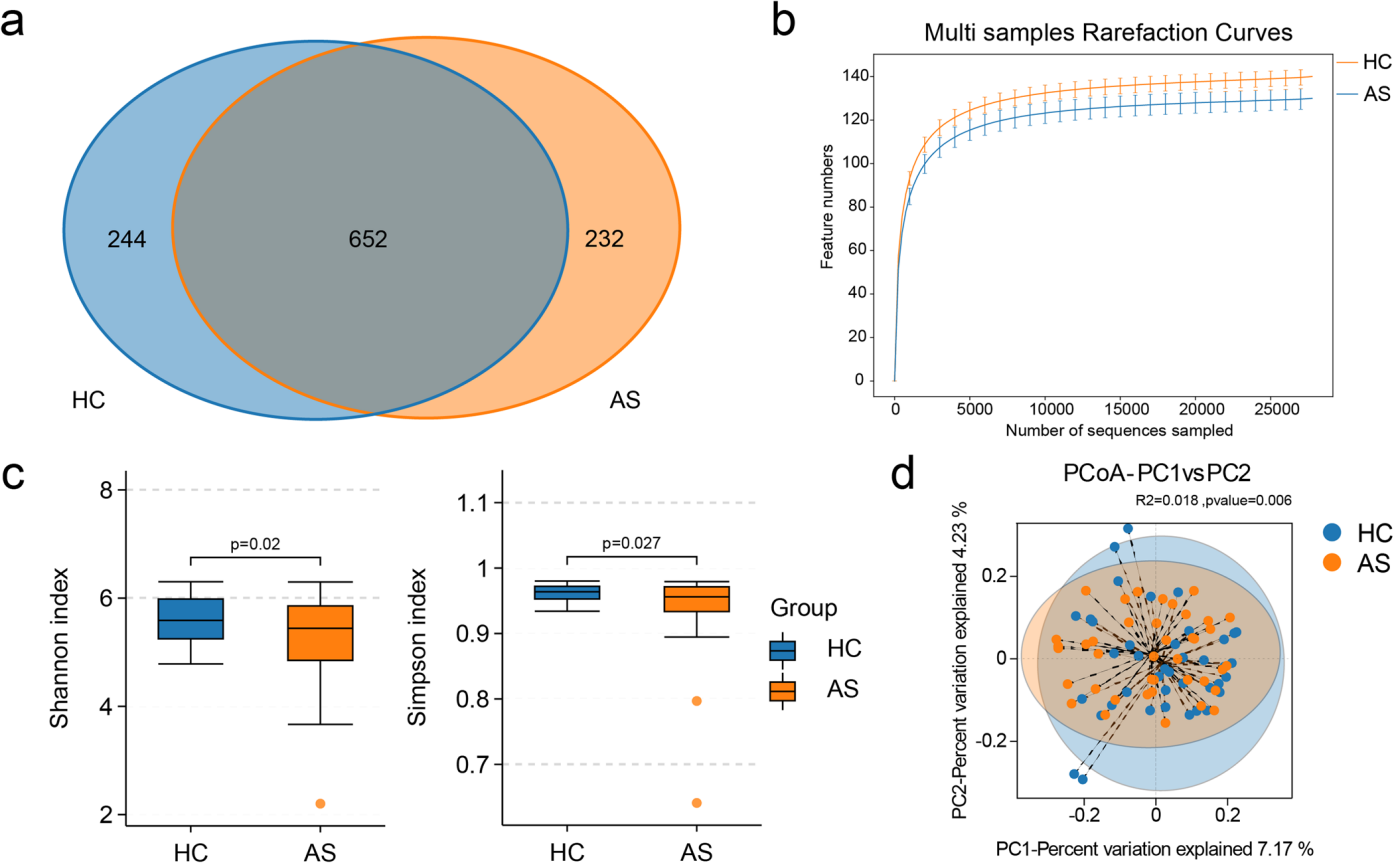
Taxonomic analyses were conducted to investigate the gut microbiota in patients with ankylosing spondylitis (AS). (a) A Venn diagram was constructed to illustrate the shared and distinct amplicon sequence variants (ASVs) between the healthy control (HC) and AS groups. (b) Rarefaction curves were generated for both the HC and AS groups to assess the sampling depth and coverage of the microbial diversity. (c) Alpha diversity analyses were performed using the Shannon diversity index and Simpson index to compare the microbial diversity between the HC and AS groups. (d) The beta diversity analysis using binary Jaccard metrics revealed a difference in the microbial composition between AS patients and HCs.

A Venn diagram illustrated the shared and unique ASVs between the AS and HC groups (Figure 1a). Among the 1128 ASVs analyzed, 652 were common to both groups. Rarefaction curves revealed that the sequencing depth was sufficient to capture the majority of the microbial information in both groups (Figure 1b).

Alpha diversity, which was assessed using the Shannon and Simpson indices, was calculated to assess the diversity of the microbial communities in the two groups. The results revealed a significant reduction in microbial diversity in AS patients compared with HCs (Figure 1c). A beta diversity analysis was employed to evaluate the similarity in microbial community composition between the two groups, aiming to identify differences in the community composition and distribution, using binary Jaccard metrics, and significant differences in the microbial composition were observed between AS patients and HCs (Figure 1d, *p* = 0.006). In summary, the species diversity of the gut microbiota in AS patients was clearly significantly lower than that in HCs.

### 3.3 Comparison of the Taxonomic and Functional Features of the Gut Microbiota Between AS Patients and HCs

**Figure 2:**
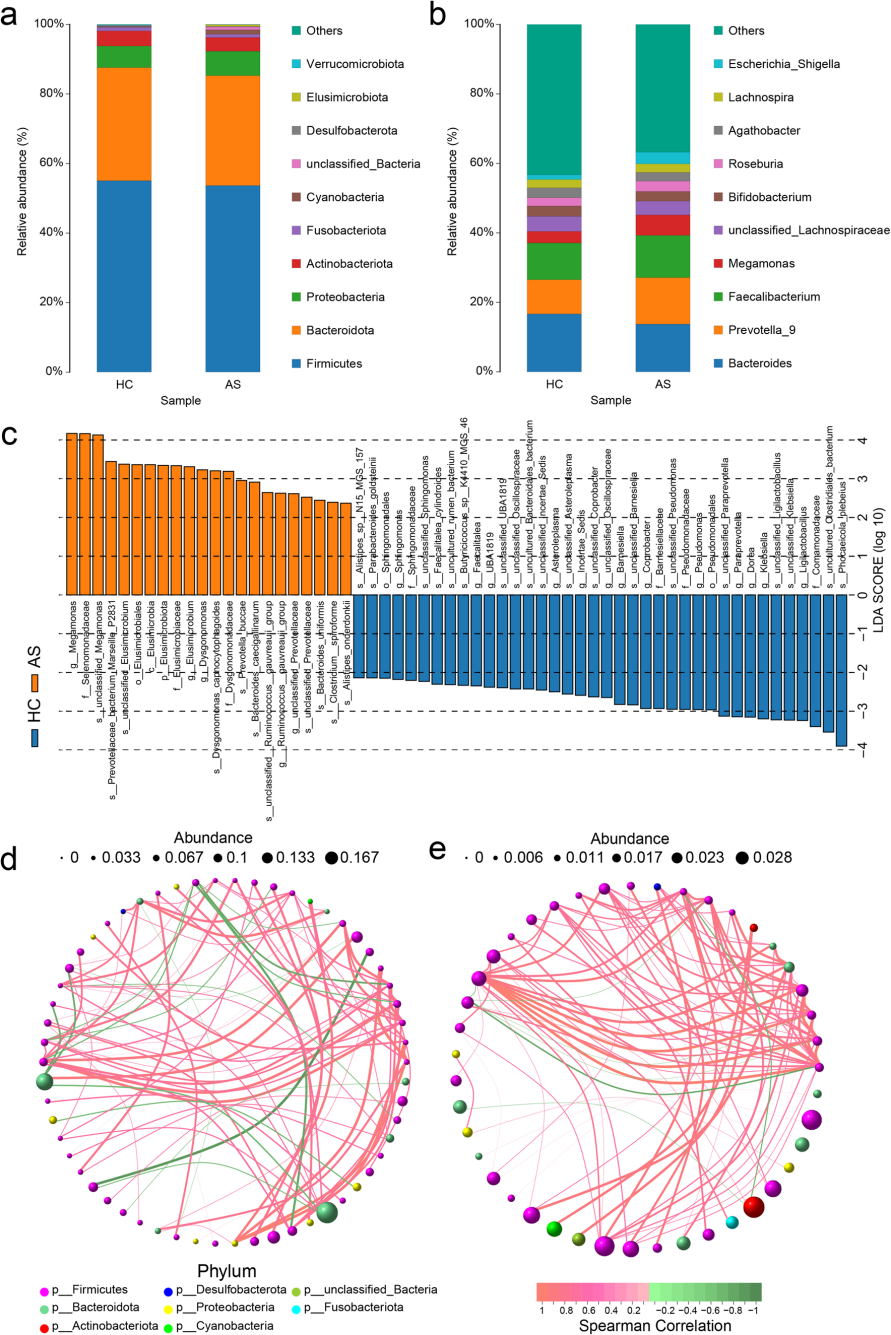
The composition and pivotal microflora in the AS and HCs groups. (a and b) At the phylum and genus level, the relative abundance of the gut microbiota was analyzed between the AS and HC groups. (c) Linear discriminant analysis effect size (LEfSe) was performed to identify the bacteria that were differentially represented between the AS and HC groups. (d and e) Network diagrams were compared using Spearman's rank correlation analysis to assess the interactions within the microbiota of the AS and HC groups.

Bar plots of the relative abundance were generated to highlight the most prevalent taxa at the phylum and genus levels. At the phylum level, *Firmicutes*, *Bacteroidota*, and *Proteobacteria* were the most abundant taxa in both the AS and HC groups (Figure 2a). At the genus level, *Bacteroides*, *Prevotella_9*, *Faecalibacterium*, and *Megamonas* dominated the gut microbiota of AS patients (Figure 2b).

A LEfSe metagenomic discovery analysis was employed using LDA to obtain additional insights into the critical microbial taxa responsible for the alteration of the gut microbiota in AS patients, and 59 taxa in the gut microbiota with significant differences in relative abundance were identified. Notably, *Megamonas, Elusimicrobium, Dysgonomonas, Ruminococcus_gauvreauii_group,* and *unclassified_Prevotellaceae* were enriched in AS patients, whereas *Ligilactobacillus, Klebsiella, Dore, Paraprevotella*, and *Pseudomonas* were more abundant in the HC group (Figure 2C).

The correlation network analysis, based on Spearman's rank correlation, revealed significant positive correlations (*r* > 0.5, *p* < 0.05) among genera in the AS group. Compared with the HC group, the AS group presented a greater density and average degree of microbial interactions, alongside lower modularity and a shorter average path length in the microbial interaction network. These results highlight the unique microbial profiles and interaction patterns associated with AS (Figure 2d,e).

A PICRUSt2 analysis based on inferred metagenomes was performed to investigate the functions of the gut microbiota in AS patients. Generally, the abundance of each pathway was similar in the two groups. Seven pathways, including glycolysis/gluconeogenesis; neomycin, kanamycin, and gentamicin biosynthesis; the MAPK signaling pathway–yeast; chloroalkane and chloroalkene degradation; porphyrin and chlorophyll metabolism; purine metabolism; and streptomycin biosynthesis, presented distinct differences in abundance between the two groups (*p* < 0.05). However, these differences were not statistically significant after adjusting for the FDR (*q* > 0.05, Benjamini‒Hochberg correction) (Table 2).

**Table 2:** Differences in metabolic pathways between AS patients and HCs.

Metabolic pathway	HC Mean ± SD	AS Mean ± SD	*p* value	95% CI
Glycolysis/gluconeogenesis	1.02 ± 0.037	0.992 ± 0.036	0.002	0.026 (0.009, 0.042)
Neomycin, kanamycin, and gentamicin biosynthesis	0.06 ± 0.008	0.055 ± 0.006	0.006	0.005 (0.001, 0.008)
MAPK signaling pathway–yeast	0.00 ± 0.005	0.003 ± 0.002	0.013	0.002 (0.000, 0.004)
Chloroalkane and chloroalkene degradation	0.07 ± 0.020	0.058 ± 0.013	0.022	0.009 (0.001, 0.016)
Porphyrin and chlorophyll metabolism	0.76 ± 0.097	0.81 ± 0.100	0.038	−0.047 (−0.091, −0.003)
Purine metabolism	2.06 ± 0.040	2.03 ± 0.074	0.042	0.028 (0.001, 0.055)
Streptomycin biosynthesis	0.3 ± 0.032	0.286 ± 0.032	0.047	0.015 (0.000, 0.029)

*Note:* This table provides a list of metabolic pathways with *p* < 0.05.

Abbreviations: AS, ankylosing spondylitis; HC, healthy control; MAPK, mitogen‐activated protein kinase; SD, standard deviation; 95% CI, 95% confidence interval.

### 3.4 Metabolomic Analysis of AS Patients Versus HCs

**Figure 3:**
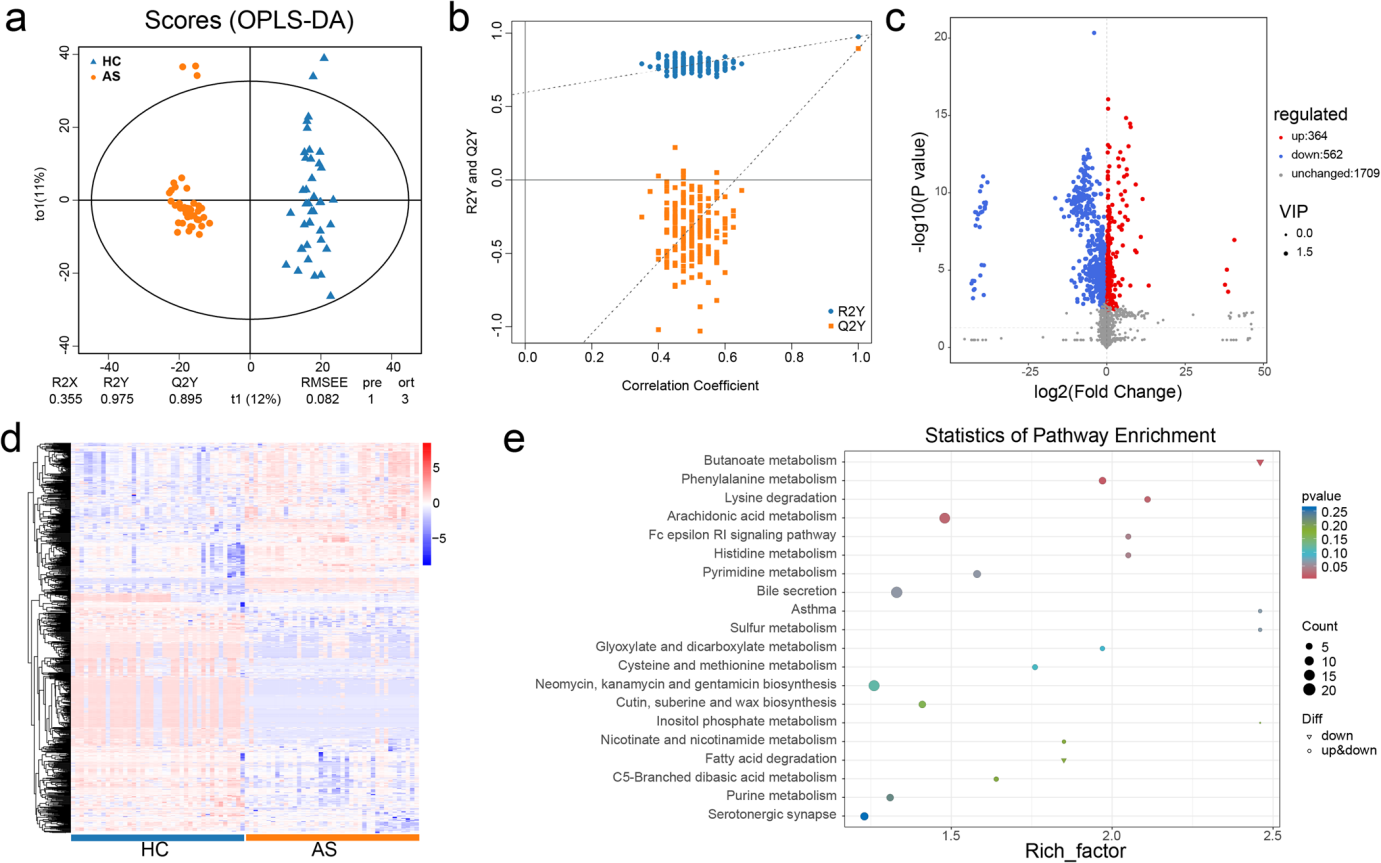
A metabolomic analysis was conducted to compare AS patients with HCs. (a) Orthogonal partial least squares discriminant analysis (OPLS‐DA) score plots were generated to visualize the differences in the metabolic profiles between the AS and HC groups. (b) Permutation plots of the OPLS‐DA models were used to validate the robustness and reliability of the model. (c) Volcano plot showing the overall trend of differences in metabolite levels between the two groups. (d) A heatmap was created to illustrate the clustering of differentially abundant metabolites, with each sample represented on the *x*‐axis and the *y*‐axis showing the standardized quantitative values of metabolite *Z* scores after the hierarchical clustering analysis. (e) An enrichment plot was generated to highlight the 20 pathways enriched with the greatest number of annotated differentially abundant metabolites.

A total of 10,507 peaks were detected and 2635 metabolites were annotated. OPLS‐DA was employed as a supervised pattern recognition method to visualize and analyze metabolic variations between AS patients and HCs (Figure 3a). The OPLS‐DA scores were significantly different between the two groups. A permutation test was conducted to validate the OPLS‐DA model, and it confirmed the reliability and robustness of the original models (Figure 3b).

Differentially abundant metabolites between the AS and HC groups were identified based on VIP scores from the OPLS‐DA model. A volcano plot was generated to provide an overview of differentially abundant metabolites and highlight statistically significant differences (*p* < 0.05) (Figure 3c). Additionally, the clustering analysis and heatmap visualization revealed clear clustering patterns of differentially abundant metabolites between the groups (Figure 3d). An enrichment analysis of these differentially abundant metabolites was performed using *clusterProfiler*, and the top 20 pathways with the greatest number of annotated metabolites were identified. The key pathways included bile secretion (*p* = 0.06); phenylalanine metabolism (*p* = 0.06); arachidonic acid metabolism (*p* = 0.02); and neomycin, kanamycin, and gentamicin biosynthesis (*p* = 0.12) (Figure 3e).

### 3.5 Correlation Analysis Between the Gut Microbiota and Metabolome in AS Patients and HCs

**Figure 4:**
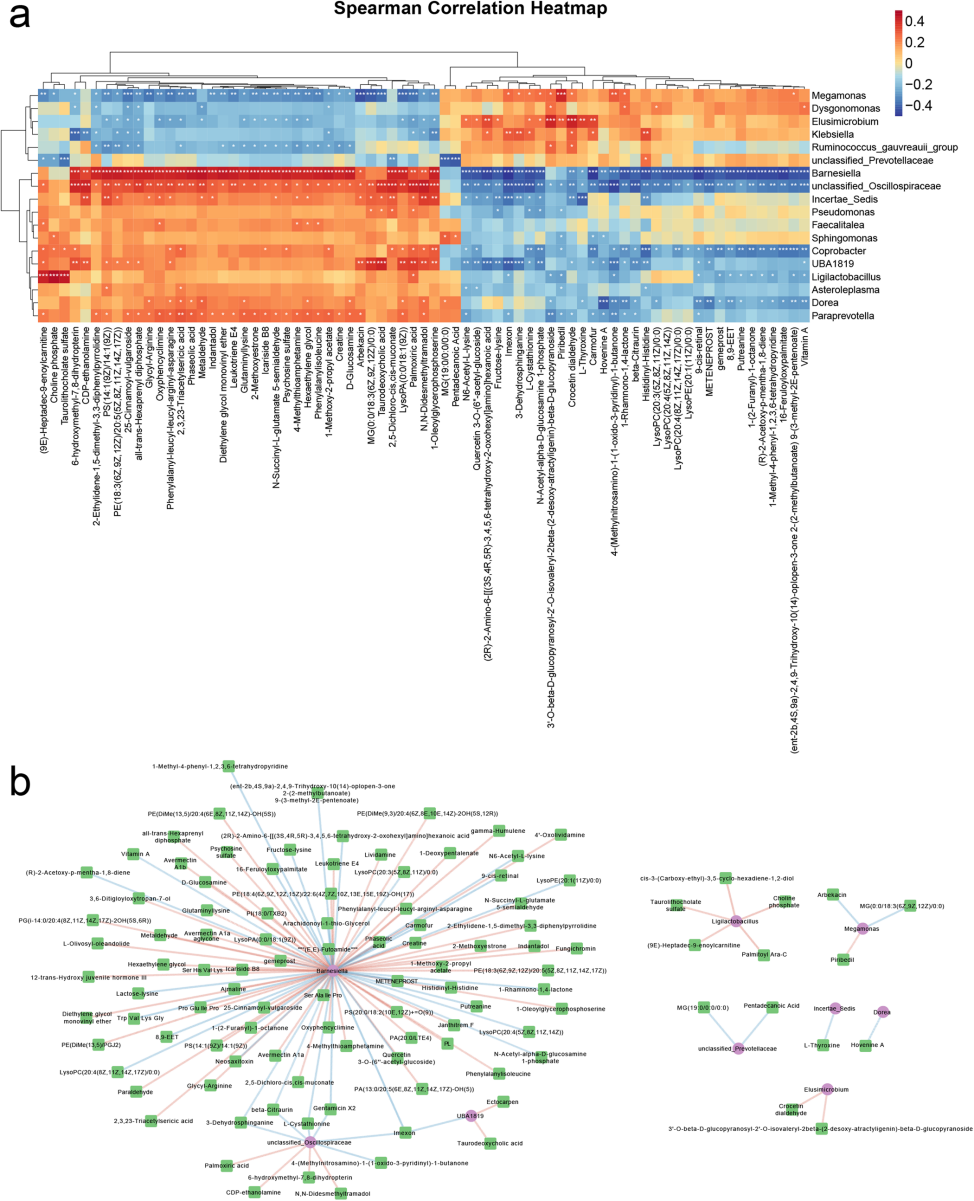
Spearman's correlation coefficients between differentially abundant genera and metabolites in the HC and AS groups. (a) Heatmaps show positive and negative correlations in red and blue, and *p* values < 0.05 are marked with asterisks. *p < 0.05, **p < 0.01, and ***p < 0.001. (b) Correlation network diagram between differentially abundant genera and metabolites. The circles in the figure represent genera, the square represent metabolites, the red lines represent negative correlations, and the blue lines represent positive correlations.

Spearman's correlation analysis was conducted to explore the associations between differentially abundant microbial genera and metabolites in the AS and HC groups (Figure 4). Strong positive correlations were observed between the abundance of several microbial genera and plasma metabolite levels. Notably, the genera *Barnesiella*, *unclassified_Oscillospiraceae*, and *Paraprevotella*, which were enriched in the HC group, presented positive correlations with multiple metabolites, including palmoxiric acid, phaseolic acid, and taurodeoxycholic acid. Conversely, *Megamonas* and *Elusimicrobium*, which were enriched in the AS group, were positively correlated with metabolites such as piribedil, l‐cystathionine, and crocetin dialdehyde. These findings suggest distinct roles for these genera and their associated metabolites, which may contribute to unique metabolite‒microbial interactions in AS and warrant further investigation.

As shown in Figure 4b, a total of 115 significant correlations between microbiota and plasma metabolites were identified based on an |*r*| > 0.4 and *p* < 0.05. Among these, *Barnesiella*, *unclassified_Oscillospiraceae*, and *Ligilactobacillus* were significantly associated with 80, 10, and 5 plasma metabolites, respectively.

### 3.6 Random Forest Diagnostic Model for the AS Group

**Figure 5:**
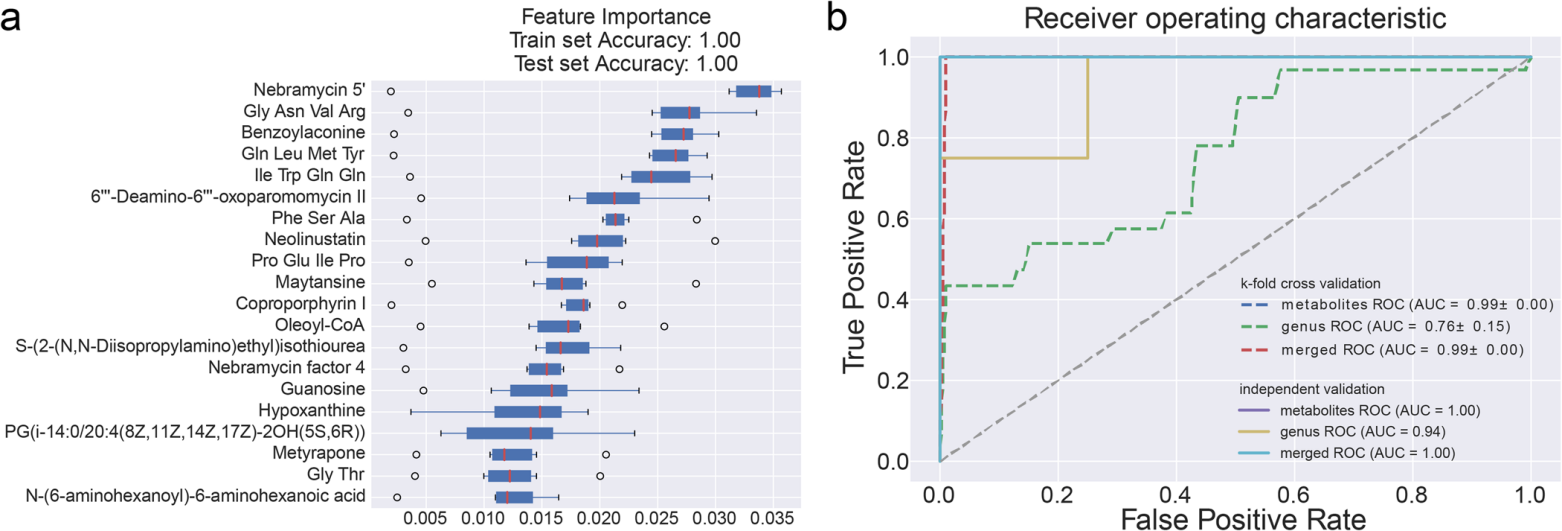
(a) Microbiota and metabolite markers for distinguishing AS patients from HCs identified from random forest classifiers based on genera and metabolites alone or the combination of the two features. (b) The performance of the classifiers was evaluated by calculating the area under the curve (AUC) via fivefold cross‐validation.

The observed differences in the gut microbiota and blood metabolites between AS patients and HCs prompted the hypothesis that a diagnostic model incorporating these profiles could enable more precise screening for AS. Random forest classifiers were developed to distinguish AS patients from HCs and to evaluate the potential of microbial and metabolic profiles as diagnostic markers. Three models were constructed: a genus‐based model, a metabolite‐based model, and a combination model (Figure 5a).

The selected taxa and metabolites differed significantly between the two groups, supporting the hypothesis that distinct biomarkers and diagnostic models are needed for AS. Using a fivefold cross‐validation strategy, the random forest classifiers demonstrated excellent predictive performance, with area under the receiver operating characteristic curve (AUC) values of 0.76, 0.99, and 0.99 for detecting AS compared with the HC group, as shown in Figure 5b.

## 4 Discussion

This study revealed significant differences in the gut microbiota composition between AS patients and HCs. A marked reduction in alpha diversity, as evidenced by decreased Shannon and Simpson indices, in AS patients indicates a disrupted intestinal environment that is associated with the disease. Furthermore, the observed increase in the abundance of 5 bacterial genera alongside a significant reduction in the abundance of 14 genera highlights an altered microbial landscape in AS patients.

Among the genera presenting an increased abundance in AS patients, *Bacteroides, Dysgonomonas, Ruminococcus, Megamonas, Elusimicrobium,* and *unclassified Prevotellaceae* were apparently enriched. Notably, *Ruminococcus*, a genus linked to SLE disease activity that is particularly enriched in lupus nephritis patients [38], exhibited a two‐ to threefold increase in AS patients, which is consistent with its established role in autoimmune diseases, especially inflammatory arthritis. These findings align with those of prior studies suggesting a correlation between *Ruminococcus* and AS disease activity, reinforcing its potential involvement in AS pathogenesis [39].

We performed Spearman's correlation analyses between the relative abundance of the gut microbiota and clinical indicators in AS patients to better understand the clinical significance of these alterations in the microbiota. The abundance of *Actinobacteriota* was positively correlated with the BASFI score, whereas the abundance of *Fusobacteriota* was positively correlated with the BASMI score. Conversely, the *Bacteroidota* abundance was negatively correlated with the BASFI. These findings are consistent with earlier reports of a decreased *Bacteroidetes* abundance in AS patients [5]. Furthermore, the *Firmicutes/Bacteroidetes (F/B)* ratio—a critical marker of intestinal homeostasis—was altered, suggesting that a shift in *Firmicutes* and *Bacteroidetes* abundances may contribute to AS pathogenesis. Such shifts in the microbial balance further highlight the intricate relationship between the gut microbiota and systemic inflammatory diseases [40, 41].

Alterations in the gut microbiota can influence host metabolic phenotypes by modulating microbial metabolites and their derivatives, such as SCFAs, lipopolysaccharides, and bile acids. In this study, the analysis of metabolic pathways revealed significant enrichment in pathways related to the biosynthesis of arginine, isoleucine, glutamine, steroid hormones, neomycin, kanamycin, gentamicin, and primary bile acids, as well as pathways linked to bile secretion and tryptophan, lipid, proline, and purine nucleotide metabolism. Arginine metabolism, for example, leads to the production of ornithine and nitric oxide, which play roles in activating innate immunity in the intestine [42, 43]. Similarly, amino acids such as isoleucine, glutamine, and tryptophan have been shown to modulate cytokine levels, microbial diversity, and arthritis severity in intervertebral joints [44].

Bile acids, which are crucial for lipid absorption and intestinal homeostasis, were also implicated in AS pathogenesis. These compounds not only aid in digestion but also influence immune pathways via receptors such as TGR5 and the PKA signaling pathway cascade, which can suppress the proinflammatory activity of macrophages [45]. Dysregulated bile acid metabolism, a feature of autoimmune diseases such as RA, SLE, and IBD [46], was similarly observed in AS patients, further supporting a shared immunopathological mechanism across these conditions [47].

Interestingly, this study identified biosynthetic pathways for antibiotics, such as neomycin, kanamycin, and gentamicin, in AS patients. Humans cannot synthesize these compounds, suggesting their origin from bacterial secondary metabolites. These secondary metabolites often mediate microbial interactions and adaptations, as evidenced by research showing that the species composition affects the expression of biosynthesis‐related gene clusters and overall metabolite profiles in microbial communities [48]. The identification of these antibiotics highlights the potential role of gut microbial dynamics in shaping the metabolic profiles of AS patients [49]. Future studies could explore how secondary bacterial metabolites modulate intestinal ecosystems and potentially influence autoimmune disease progression.

Despite these findings, the causal relationship between microbial metabolites and AS pathogenesis remains speculative [50]. The observed metabolic changes may plausibly either drive disease development or reflect underlying immunopathological alterations. Further mechanistic studies are needed to establish these links definitively.

Several limitations should be acknowledged. First, the relatively small sample size limits the generalizability of our findings. Second, the cross‐sectional nature of this study precludes a causal inference, and longitudinal analyses are necessary to investigate how the gut microbiota and metabolic profiles change during treatment or disease remission. Third, our analysis relied on plasma samples, and while correlations between specific bacteria and metabolites were identified, these associations may also be influenced by external factors such as the diet. Moreover, the use of NSAIDs by patients in this cohort could have altered the microbial composition and metabolite profiles, potentially confounding our results. As a method to address this limitation, we designed this study to minimize the confounding effects of NSAIDs. Specifically, all AS patients in our cohort had undergone similar NSAID treatment, ensuring that any observed differences between the AS and HC groups were more likely attributable to the disease itself rather than to variability in treatment. Additionally, we controlled for other potential confounders, such as age and sex, in our analysis (Table 1). Despite these measures, we acknowledge that NSAID treatment remains a potential confounding factor that may have influenced the microbiota and metabolite profiles observed in AS patients. This limitation has been highlighted to ensure transparency and is an important area for future research. Future studies should control for these variables and include untreated patients or those receiving diverse therapeutic interventions.

## 5 Conclusions

In summary, this study employed integrative fecal microbiome and plasma metabolomic analyses to investigate the relationships among the gut microbiota, metabolites, and AS pathogenesis. Significant alterations in both the microbial composition and metabolic pathways were observed in AS patients compared with HCs. These findings highlight that a disrupted gut microbiota and altered metabolic signatures are associated with AS, providing valuable insights into the interplay among the intestinal microbiota, metabolites, and autoimmune diseases. Further research is needed to elucidate these interactions and explore their therapeutic potential in AS management.

## Author Contributions

Conception: Xin Wang and Haojie Xu; methodology: Haojie Xu, Yuyan Chao, Lin Tang, Changhao Xie, and Shengqian Xu; data curation: Haojie Xu, Xin Wang, and Xiaoyun Fan; analysis: Haojie Xu and Yuyan Chao; interpretation and writing: Haojie Xu, Yuyan Chao, and Tingting Wang; supervision: Changhao Xie and Shengqian Xu. All authors were involved in drafting this paper or revising it critically for key intellectual content, and all authors approved this version to be published. Dr. Shengqian Xu and Dr. Changhao Xie had full access to all the data in the study and take responsibility for the integrity of the data and the accuracy of the data analysis.

## Ethics Statement

The study was approved by the Institutional Review Board of Bengbu Medical University (Ethics Review No. 2023‐073).

## Consent

Written informed consent was obtained from all participants.

## Conflicts of Interest

The authors declare no conflicts of interest.

## Data Availability

The raw sequencing data generated in this study have been deposited in NCBI Sequence Read Archive (http://www.ncbi.nim.nih.gov/sra) under the Accession Number PRJNA1032265.
